# Cardiac sarcoidosis resembling panic disorder: a case report

**DOI:** 10.1186/s12888-016-1184-z

**Published:** 2017-01-13

**Authors:** Keita Tokumitsu, Jun Demachi, Yukichi Yamanoi, Shigeto Oyama, Junko Takeuchi, Koji Yachimori, Norio Yasui-Furukori

**Affiliations:** 1 Department of Neuropsychiatry, Towada City Hospital, Towada, 034-0093 Japan; 2 Department of Cardiology, Towada City Hospital, Towada, 034-0093 Japan; 3 Department of Neuropsychiatry, Graduate School of Medicine, Hirosaki University, Hirosaki, 036-8562 Japan

**Keywords:** Cardiac sarcoidosis, Panic disorder, Panic attacks, Anxiety disorder

## Abstract

**Background:**

Sarcoidosis is a systemic disease of unknown etiology, in which granulomas develop in various organs, including the skin, lungs, eyes, or heart. It has been reported that patients with sarcoidosis are more likely to develop panic disorder than members of the general population. However, there are many unknown factors concerning the causal relationship between these conditions.

**Case presentation:**

We present the case of a 57-year-old woman who appeared to have panic disorder, as she experienced repeated panic attacks induced by transient complete atrioventricular block, associated with cardiac sarcoidosis. Psychotherapy and pharmacotherapy were not effective in the treatment of her panic attacks. However, when we implanted a permanent pacemaker and initiated steroid treatment for cardiac sarcoidosis, panic attacks were ameliorated. Based on these findings, we diagnosed the patient’s symptoms as an anxiety disorder associated with cardiac sarcoidosis, rather than panic disorder.

**Conclusions:**

This report highlights the importance of considering cardiac sarcoidosis in the differential diagnosis of panic disorder. This cardiac disease should be considered especially in patients have a history of cardiac disease (e.g., arrhythmia) and atypical presentations of panic symptoms. Panic disorder is a psychiatric condition that is typically diagnosed after other medical conditions have been excluded. Because the diagnosis of sarcoidosis is difficult in some patients, caution is required. The palpitations and symptoms of heart failure associated with cardiac sarcoidosis can be misdiagnosed as psychiatric symptoms of panic disorder. The condition described in the current case study appears to constitute a physical disease, the diagnosis of which requires significant consideration and caution.

## Background

Panic disorder is characterized by repeated panic attacks and anticipatory anxiety concerning these attacks [1]. Importantly, exclusion of other physical diseases is required for a diagnosis of panic disorder [1]. Because panic attacks may be caused by physical diseases, such as hyperthyroidism, brain tumors, or arrhythmias, panic disorder should be diagnosed with caution [1].

Sarcoidosis is a systemic disease of unknown etiology in which granulomas develop in various organs (e.g., the skin, lungs, eyes, or heart) [2]. Cardiac lesions are specifically referred to as cardiac sarcoidosis, a condition that can lead to fatal arrhythmias or heart failure [2]. It has been reported that 44% of patients with sarcoidosis experience comorbid psychiatric disorders, with panic disorder observed in 6.3% of cases [3]. The prevalence of panic disorder among patients with sarcoidosis is higher than that of the 2.7% reported in the general population [4]. However, many aspects of the causal relationship between these conditions are currently unknown.

## Case presentation

**Fig. 1:**
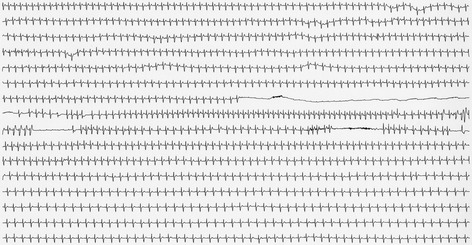
The onset of panic attack symptoms was consistent with electrocardiographic abnormalities. A 24-h ambulatory electrocardiogram revealed cardiac pause (maximal 32 s) due to a complete atrioventricular block, and transient sinus tachycardia following recovery from the pause. The onset of panic attack symptoms was consistent with these electrocardiographic abnormalities

We present the case of a 57-year-old woman. The patient visited our emergency room because of sudden palpitations and dyspnea. Physical examination revealed stable and sustained ventricular tachycardia. However, ventricular tachycardia returned spontaneously to a normal sinus rhythm. After admission to the cardiology ward, further examinations, including left heart catheterization, were performed. However, the cause of ventricular tachycardia remained unclear. Following discharge, the patient experienced palpitations and dyspnea approximately once a month. Therefore, we repeatedly performed a 12-lead electrocardiogram, a 24-h ambulatory electrocardiogram, hematologic examinations, and chest radiography. These examinations revealed no clear abnormalities that could explain the palpitations. It was suspected that the patient’s physical symptoms were due to a psychiatric condition. Thus, one and a half years after her initial visit to the cardiology ward, she was referred to the department of neuropsychiatry. The patient experienced palpitations and dyspnea at rest without any apparent cause. These symptoms sometimes continued for two to three hours. During each incident, the patient experienced a strong fear of dying. She also experienced strong anticipatory anxiety and left her house less frequently than she used to because of the fear that palpitations would occur. Hematologic findings revealed that the patient’s thyroid hormone levels were within the normal range. Furthermore, cranial computed tomography findings indicated no clear abnormalities. Because no structural abnormalities were noted and the patient was experiencing repeated panic attacks, we diagnosed her symptoms as panic disorder. After assuring the patient that panic attacks are not fatal, we attempted cognitive behavioral therapy and pharmacotherapy (alprazolam 1.2 mg/day and mirtazapine 15 mg/day). However, the panic attacks continued. The frequency of panic attack symptoms progressively worsened until she experienced daily panic attacks. Approximately 2 months after her initial visit to the department of neuropsychiatry, a 24-h ambulatory electrocardiogram revealed cardiac pause (maximal 32 s) due to a complete atrioventricular block, and transient sinus tachycardia following recovery from the pause (Fig. 1). Moreover, the onset of panic attack symptoms was consistent with these electrocardiographic abnormalities. Therefore, a permanent pacemaker was implanted. Following implantation of the permanent pacemaker, the frequency of the panic attacks improved, and anticipatory anxiety and fear of death disappeared. However, 1 month after implantation of the permanent pacemaker, the patient was brought to our hospital’s emergency department with symptoms of heart failure. Echocardiographic findings revealed a decrease in left ventricular systolic function (ejection fraction 40%) and basal thinning of the interventricular septum. Thus, cardiac sarcoidosis was suspected. Serum angiotensin-converting enzyme and soluble interleukin-2 receptor levels were within reference ranges, but gallium scintigraphy revealed significant gallium uptake in the heart. We did not perform a myocardial biopsy because of the risk of cardiac perforation, cardiac tamponade and dislodging the pacemaker leads. Based on the diagnostic criteria for cardiac sarcoidosis [5], we made a clinical diagnosis of cardiac sarcoidosis. Other clinical findings suggested that the patient also had concomitant pulmonary sarcoidosis, as extracardiac sarcoidosis. Chest X-ray findings did not reveal any clear bilateral hilar lymphadenopathy, but a chest computed tomography scan revealed multiple nodular shadows suggestive of sarcoid granulomas. Furthermore, gallium scintigraphy revealed significant gallium uptake in the lung. No findings suggestive of skin manifestations of sarcoidosis or neurosarcoidosis were observed. We started steroid therapy (prednisolone 30 mg/day), and the patient’s cardiac function and gallium scintigraphic abnormalities were ameliorated. This improvement indicated that the cause of complete atrioventricular block was associated with cardiac sarcoidosis. Therefore, the set of symptoms that were initially attributed to panic disorder were actually caused by a complete atrioventricular block associated with cardiac sarcoidosis, which secondarily caused panic attacks. The patient was subsequently diagnosed with an anxiety disorder associated with cardiac sarcoidosis, rather than panic disorder.

## Discussion

In the case described in this report, differentiating cardiac sarcoidosis from panic disorder was difficult. According to the DSM-5, the diagnosis of panic disorder is applied in cases of repeated and unexpected panic attacks, where the condition is defined as not being caused by the physiological effect of a substance or other medical condition (e.g., hyperthyroidism or a cardiopulmonary disorder) [1]. Panic attacks are psychiatric symptoms in which sudden severe fear or discomfort increases and peaks within a few minutes, and four of 13 symptoms (palpitations, sweating, trembling, sensations of shortness of breath, feelings of choking, chest pain, nausea, dizziness, chills, paresthesia, derealization, fear of losing control, or fear of dying) occur during that time [1]. In the case described in the current report, the patient exhibited palpitations, dyspnea, chest pain, and fear of death. Furthermore, the patient experienced anticipatory anxiety of panic attacks, and because of the fear of future attacks, the frequency with which she was able to leave her house decreased. Although the patient had a history of one episode of ventricular tachycardia, an outpatient cardiovascular examination revealed no structural abnormalities to explain the repeated palpitations, and the patient was referred to the department of psychiatry. The examination indicated that the patient’s thyroid function was normal, and no other structural abnormalities were noted at the initial visit. Therefore, we assumed that the palpitations were due to panic disorder. Thus, we initiated psychotherapy and the administration of psychotropic medication, but observed no therapeutic effects. During the clinical course, it became clear that the cause of panic attacks was complete atrioventricular block associated with cardiac sarcoidosis and transient sinus tachycardia following recovery from the cardiac pause. Because the attacks were not frequent and occurred approximately once a month, it was difficult to detect the electrocardiographic abnormalities using a 24-h ambulatory electrocardiogram, which may have contributed to the delay in the diagnosis. To perform an accurate diagnosis earlier, it may have been useful to use an implantable loop recorder to analyze electrocardiographic findings during attacks [6]. Although our patient was inserted a permanent pacemaker to treat complete atrioventricular block, it was initially not possible to diagnose her with cardiac sarcoidosis. Furthermore, ventricular tachycardia was only noted once during the initial presentation, and its relationship with the subsequent cardiac sarcoidosis was unclear. However, considered retrospectively, the ventricular tachycardia and complete atrioventricular block might have appeared as a series of symptoms caused by cardiac sarcoidosis. When a treatment plan is determined, implantation of a dual chamber pacemaker/defibrillator should be considered to treat recurrence of ventricular tachycardia [7, 8]. However, the possibility of depression, anxiety, or other emotional impact that could result from ICD implantation should also be considered [9], especially because our patient had a history of panic attacks and anxiety disorder. We suggest that sufficient patient-physician communication is important before ICD implantation [9].

An increasing number of recent studies have reported a relationship between sarcoidosis and psychiatric disorders. In the general population, the prevalence rate of panic disorder is reportedly 2.7% [4]. By contrast, Goracci et al. reported that 6.3% of patients with sarcoidosis also exhibit panic disorder, and 25% of patients with cardiac sarcoidosis also have depression, while 5% also have an anxiety disorder [3]. A possible relationship between the decrease in respiratory function and psychiatric symptoms (e.g., anxiety disorder and depressive disorders) has been suggested [3]. But there are many unknown factors concerning the causal relationship between the conditions. Psychiatric manifestations, including apathy, delirium and depression, occur in about 20% of patients with neurosarcoidosis, reflecting the potential for granulomatous infiltration of any part of the central nervous system [10]. In a study examining paroxysmal supraventricular tachycardia, Colucci et al. noted that some patients receiving a delayed diagnosis of arrhythmia are initially misdiagnosed as having panic disorder [11]. As observed in the present case, it is possible for patients with arrhythmia associated with cardiac sarcoidosis causing panic attacks in whom physical diseases have been overlooked to be misdiagnosed with panic disorder. However, no previous reports have highlighted the importance of differentiating cardiac sarcoidosis from panic disorder.

In cases of cardiac sarcoidosis, in conjunction with the progression of the disease, clinical symptoms, such as dizziness, syncope, palpitations, and dyspnea, are typically observed due to cardiac dysfunction. However, because these symptoms are nonspecific and the progression of the disease is slow, diagnosis is often delayed. According to autopsy reports of patients with sarcoidosis, cardiac involvement is present in at least 27% of cases [12], but cardiac sarcoidosis is clinically observed in only 5% of patients [13]. This finding indicates that many cardiac lesions are overlooked ante mortem [2, 14]. In our case, echocardiography revealed basal thinning of the interventricular septum, a characteristic feature of cardiac sarcoidosis, which served as the steppingstone that led to the accurate diagnosis of this condition [15, 16]. Thus, echocardiography should be considered in patients who develop new-onset panic attacks at an older age as panic disorder or in individuals with a history of arrhythmias or other cardiac problems that could lead to panic-like symptoms.

Although sarcoidosis spontaneously subsides over time in some patients, caution is required, as fatal cases of arrhythmia and severe heart failure may develop in patients with cardiac involvement. Histological diagnosis of cardiac sarcoidosis requires myocardial biopsy, but diagnostic sensitivity is low (19.2%) [17]. Thus, it is necessary to consider early steroid treatment based on the clinical diagnosis when cardiac sarcoidosis is clinically suspected [18, 19].

Because panic disorder is a psychiatric disorder in which diagnosis is contingent on excluding other physical symptoms, in the case of indolent diseases, accurate differentiation during the initial stages of the disorder is difficult. Therefore, it is important to differentiate physical diseases when psychiatric treatment initiated under the diagnosis of panic disorder is ineffective.

## Conclusions

The current case report highlights the importance of considering cardiac sarcoidosis in the differential diagnosis of panic disorder. This cardiac disease should be considered especially the patients have a history of cardiac disease (e.g., arrhythmia) and atypical presentations of panic symptoms.

Panic disorder is a psychiatric disorder that is diagnosed after other medical conditions have been excluded. Because differentiation is difficult in some patients, caution is required. Moreover, it is possible for palpitations and heart failure symptoms associated with cardiac sarcoidosis to be misdiagnosed as psychiatric symptoms of panic disorder. Therefore, the condition described in the present report appears to constitute a physical disease for which caution is required in psychiatric diagnosis.
